# Energy-efficient priority encoding strategies using machine learning based hybrid MAC protocol for wireless sensor networks

**DOI:** 10.1038/s41598-025-31752-1

**Published:** 2025-12-22

**Authors:** Nasser S. Albalawi, Yazeed Alzahrani, Nada Alsalmi, Yogesh Patidar, Manoj Tolani

**Affiliations:** 1 Department of Computer Sciences, Faculty of Computing and Information Technology, Northern Border University, Rafha, 91911 Saudi Arabia; 2 Department of Computer Engineering and Information, College of Engineering in Wadi Addwasir, Prince Sattam bin Abdulaziz University, Wadi Addwasir, Saudi Arabia; 3 Department of Computer Science and Artificial Intelligence, College of Computer Science and Engineering, University of Jeddah, Jeddah, Saudi Arabia; 4 Department of Electronics and Telecommunications Engineering, Government Polytechnic College Mandsaur, Mandsaur, Madhya Pradesh 458001 India; 5 Department of Electronics and Communication Engineering, Jaypee Institute of Information Technology, Noida, Uttar Pradesh 201309 India

**Keywords:** Medium access control, Priority data transmission, Periodic data transmission, Priority encoding, Latency, Computer science, Electrical and electronic engineering

## Abstract

In the present work, a Priority-Aware Periodic Hybrid MAC protocol ($Pa^2HMAC$) is proposed to achieve intelligent, energy-efficient, and priority-based data transmission in Wireless Sensor Networks (WSNs). WSNs frequently encounter challenges in efficiently managing heterogeneous traffic—such as event-driven, periodic, and emergency data—while maintaining low latency and reduced energy consumption. Traditional MAC protocols rely on static scheduling, lacking the adaptability needed to handle critical or time-sensitive data under varying network conditions, leading to packet delays, energy wastage, and reduced responsiveness. The proposed $Pa^2HMAC$ protocol integrates a machine learning-based priority encoding mechanism that evaluates data transmission priorities based on three key factors: data priority, emergency data, and buffer overflow. It operates in two distinct modes—Normal and Priority—to dynamically adapt to changing network requirements. In Normal mode, sensor nodes transmit data using either TDMA or BMA schemes depending on traffic type, while the central controller optimally determines sampling rates for periodic data. When emergency or high-priority conditions occur, the system switches to Priority mode to ensure timely and reliable data delivery. Simulation results demonstrate that the proposed $Pa^2HMAC$ outperforms existing TDMA, EA-TDMA, EBMA, and ASHMAC protocols in terms of energy efficiency, and latency reduction, thereby offering a robust framework for priority-aware, energy-efficient communication in WSNs.

## Introduction

Efficient Medium Access Control (MAC) protocol is a primary requirement and plays a critical role in managing robust communication, energy consumption, and data transmission reliability in Wireless Sensor Networks (WSN)^1^. The traditional MAC protocols, such as Time Division Multiple Access (TDMA)^2^ and its variants e.g. Bit Map Assisted (BMA)^3^, Energy efficient BMA (E-BMA)^4^, have been reported and widely used due to their less complexity and ability to manage different types of data traffic, such as event-driven^5,6^ and continuous transmissions^7–9^. The researchers have reported several works, and significant research efforts have contributed to enhance these protocols^10–13^. The primary focus of the researchers is to focus on specific areas like improving energy efficiency^4,14^, efficient bandwidth utilization^15^, and ensuring guaranteed timely delivery of priority and emergency data^16^. The advancement reported in TDMA-based protocols e.g., Energy efficient-TDMA (E-TDMA)^2^ and Application Specific Hybrid MAC (ASHMAC)^17^ have used to optimize the time slot allocation and reduce energy consumption, while protocols like EBMA^4^ have aimed to improve channel utilization and reliability but it compromises with delay.

However, the existing methods face various limitations in scenarios involving mixed data traffic patterns and priority-based data transmissions^16^. Specifically, they often fail to account for periodic data transmission, varying data priorities^6^, such as emergency data, buffer overflows, or time-sensitive information^18,19^. In the existing methods, the ideas are proposed but the proposed methods either compromises with energy consumption or latency^17,20^. In addition, existing methods adopts static slot allocation schemes that lack flexibility in adapting to dynamic network conditions^16^, which can result in inefficient resource utilization and wastage of energy consumption as well as bandwidth. The complexity and the scale of the WSN is continuously increasing, particularly for fields like healthcare monitoring^6^, industrial automation^9^, and environmental surveillance^10^, there is a need for more adaptive and priority-aware energy efficient and guaranteed delay communication protocols that can address these challenges.

One of the major challenges in WSNs is ensuring that high-priority or emergency data^21^ is transmitted promptly, without causing significant delays to regular data traffic or compromising energy efficiency^17,22^. The existing static slot allocation methods often lead to resource contention and buffer overflows, which can delay the transmission of critical data. Also, dynamic slot allotment methods like Adaptive Bit Map Assisted (ABMA)^23^ fails to allot guaranteed time slot.The ABMA and ASHMAC^17^ perform well for purely continuous and event data traffic conditions. Moreover, continuous traffic streams, which include both purely continuous and periodic data, require more refined slot allocation techniques to prevent congestion and optimize energy use. Another challenge arises from the heterogeneity of data traffic in WSNs, where event-driven and continuous transmissions need to be handled differently to ensure balanced resource utilization^22^.

To address the above discussed challenges, the present work proposes a priority-aware periodic hybrid MAC protocol, denoted as $Pa^2HMAC$. The proposed method introduces a more precise slot allocation mechanism based on data priority, emergency status, and buffer conditions. A machine learning-based priority encoding method is proposed which adapts dynamically to the network’s needs, ensuring efficient data transmission. By precisely estimating the network need , the protocol either enables normal mode or priority mode. In Normal mode, sensor nodes transmit data based on traffic type, with optimal sampling rates for periodic data traffic determined by a central device. For critical conditions, such as buffer overflow or emergency data, are detected, the protocol switches to Priority mode to prioritize urgent data transmission.

The contribution and novelty of the proposed method is discussed below:

- The proposed $Pa^2HMAC$ introduces a hybrid MAC protocol that operates in two distinct modes—Normal mode and Priority mode—allowing for dynamic adaptation to varying data traffic priorities, including emergency data and buffer overflow scenarios.
- For the priority mode, a priority encoding method is proposed and formulated to efficiently utilize the channel for the needy nodes. The protocol uniquely prioritizes emergency and time-sensitive data transmission by switching to Priority mode when critical conditions are detected, ensuring prompt and reliable data delivery without compromising normal traffic.
- A machine learning-based priority encoding method is employed to optimize slot allocation, enhancing the protocol’s ability to handle mixed traffic types (event-driven, continuous, and periodic) more efficiently than static approaches.
- The mathematical model of the proposed approach is developed to improve energy efficiency by refining slot allocation based on traffic type and priority. The analytical model ensures that the proposed approach optimizes resource utilization, particularly for periodic data, and reduces unnecessary energy consumption compared to conventional TDMA-based protocols and their variants.
- The $Pa^2HMAC$ protocol has been evaluated against established protocols like TDMA, EA-TDMA, EBMA, and ASHMAC, demonstrating superior performance in terms of energy consumption, and latency.

The rest of the paper is organized as described in this section. Section 3 presents methodology of the proposed method. Section 4 outlines the mathematical modeling and algorithm of the proposed research work. In Sect.  5, the results are described considering various scenarios and evaluating metrics, including transmission probability, number of rounds, packet size, and network density. In addition, the performance of the proposed method is compared with the performance of the existing methods, i.e., TDMA, EA-TDMA, EBMA, and ASHMAC. Finally, Sect.  6 concludes the paper, summarizing the main findings and discussing future research directions.

## Related works

Over the years, numerous MAC protocols have been developed for wireless sensor networks (WSNs), aiming to balance energy efficiency and data transmission reliability. Protocols such as EBMA^4^ and EATDMA^2^ provided foundational TDMA-based scheduling schemes but lacked dynamic adaptability to varying traffic types. ABMA^23^ improved upon this by considering periodic traffic and enhanced slot reuse, while ASHMAC^17^ introduced bitmap-assisted scheduling for better delay and fairness handling. Similarly, BEST-MAC^20^ and VTA-SMAC^18^ proposed slot contention mechanisms with energy-saving features, but were not equipped to handle heterogeneous or critical traffic prioritization. Despite offering high energy efficiency, many of these protocols underperformed in network utilization and fairness under mixed or congested traffic conditions. Recent studies have explored machine learning-based enhancements in MAC and resource allocation frameworks. For instance, Maurya et al.^24^ present a detailed survey on data-driven MAC strategies in LoRaWAN networks, highlighting the growing role of ML in scalable IoT environments. Similarly, Gupta et al.^25^ propose reinforcement learning-based resource allocation for vehicular cognitive radio networks, demonstrating the adaptability of ML in next-generation MAC and spectrum control systems. Several recent works have addressed energy-efficiency in sensor networks under specialized contexts. Hussain et al. propose an energy-efficient synchronization scheme for body sensor networks (EESBSN) that reduces path loss and balances energy consumption via dual synchronization and intelligent node selection^26^. Similarly, in underwater sensor networks, Altaf et al. introduce a trust-based, void-hole avoidance protocol (EETAUV) that integrates localization and trust management for energy and stability gains in 6G-enabled UASNs^27^.

Recent advancements in intelligent wireless communication have used machine learning to optimize network adaptability and energy efficiency. Zhang et al.^28^ introduced a gradient compression and correlation-driven federated learning framework for wireless traffic prediction, improving scheduling and communication efficiency in dense networks. Mehmood et al.^29^ proposed a QoS-based multi-path routing scheme for smart healthcare monitoring in body area networks, emphasizing energy-aware data transmission and reliability under mixed traffic conditions.

Recent application-specific protocols such as the MAC for Greenhouse monitoring^12^, MAC for Linear Networks^13^, and OMAC for WBAN^15^ focused on constrained environments, optimizing for domain-specific requirements. However, their generalizability to diverse, real-time scenarios is limited. The comparative analysis is shown in Table 1. From the comparative summary, it is evident that while some protocols excel in energy efficiency (e.g., ASHMAC, OMAC), others struggle with fairness, channel contention, or data optimization.

To bridge these gaps, the proposed PaHMAC and $Pa^2HMAC$ strategies utilize a lightweight machine learning-based prioritization to dynamically classify and manage emergency, priority, and periodic traffic. By introducing an intelligent control mechanism that adapts slot scheduling and transmission modes, the proposed approach significantly improves delay performance, energy-efficiency, and network utilization—resolving several key limitations observed in existing MAC protocol designs.

**Table 1:** Comparative overview of various MAC Protocols.

Protocol	Year	F	DLY	EFF	NU	PERF	CONT	DO
ASHMAC^17^	2020	✓	✓	✓	✗	✓	✗	✗
EBMA^4^	2013	✗	✓	✓	✗	✓	✗	✗
EATDMA^2^	2008	✗	✓	✓	✗	✓	✗	✗
ABMA^23^	2019	✗	✓	✓	✗	✓	✓	✓
MAC for Greenhouse^12^	2023	✗	✓	✓	✗	✗	✗	✓
MAC for Linear Networks^13^	2022	✗	✓	✓	✗	✗	✓	✗
OMAC (WBAN)^15^	2022	✓	✓	✓	✗	✓	✓	✗
BEST-MAC^20^	2016	✗	✓	✓	✗	✗	✓	✗
VTA-SMAC^18^	2021	✗	✓	✓	✗	✗	✓	✗

Note: **F** = Fairness, **DLY** = Delay, **EFF** = Energy Efficiency, **NU** = Network Utilization, **PERF** = Performance, **CONT** = Channel Contention, **DO** = Data Optimization

## Methodology of the proposed model

**Fig. 1:**
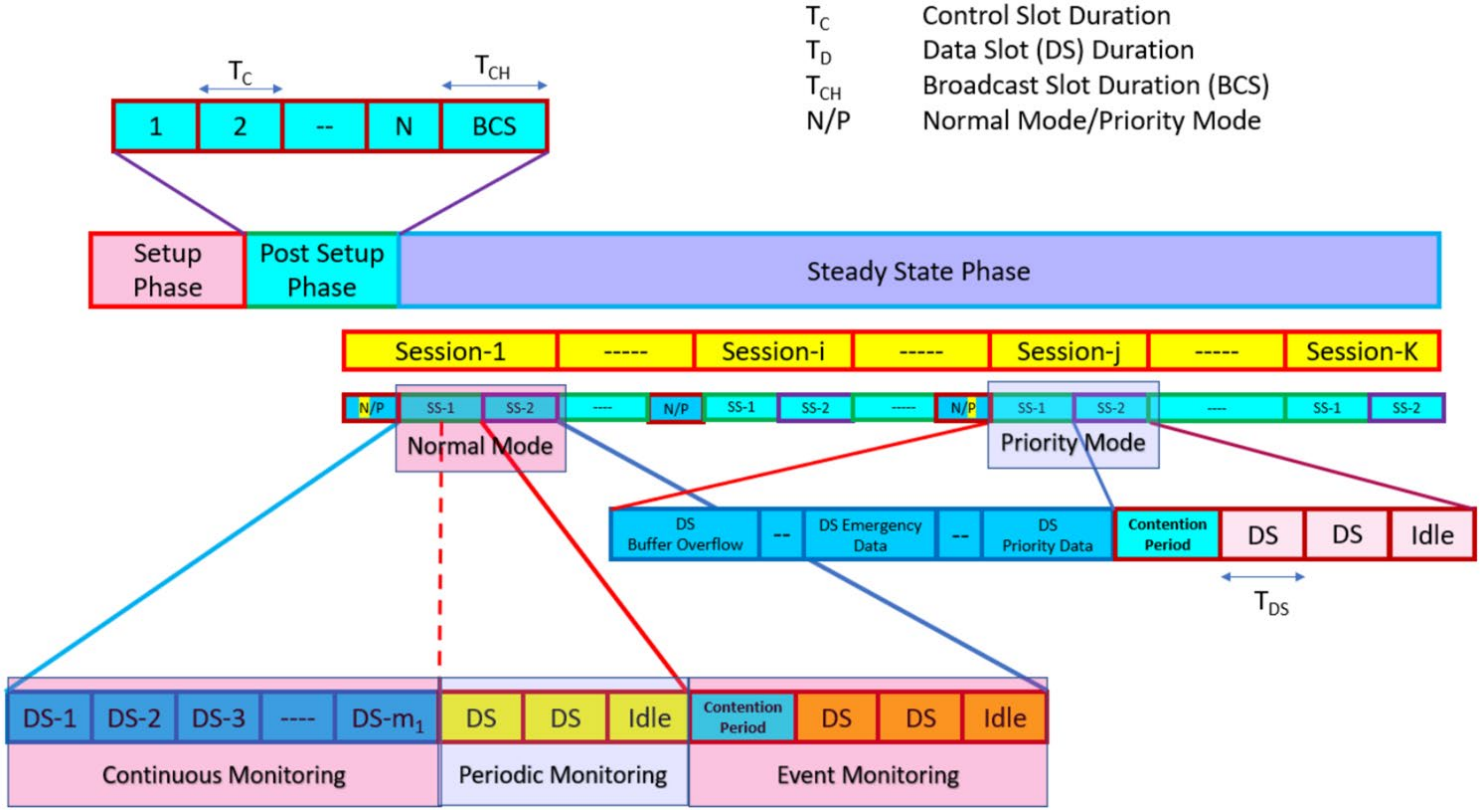
Frame architecture of the proposed protocol.

As shown in Fig.1, the proposed method operates in two main phases, i.e., setup phase and the steady state phase. The setup phase initializes the system, while the post setup phase transitions the system into its steady state. In post set-phase, the devices are categorized in continuous/periodic/event monitoring nodes. During the Steady State Phase, various sessions take place, divided into control, data, and broadcast slots.

Within the steady state phase, the system can switch between Normal Mode and Priority Mode. In Normal Mode, regular monitoring and data transmission takes place from Sensor Node (SN) to Cluster Head (CH) node. The system monitors various activities (e.g. temperature, humidity, etc.) continuously or periodically based on the type of sensor and operation requirement during normal operations. There are multiple sessions (Session 1 to Session K) represented in this phase. Each session is divided into two sub-sessions. The session starts with broadcasting of control message for the selection of mode based on the network requirement. For normal operation, the traditional TDMA method is used for continuous monitoring nodes during sub-session-1 and for periodic monitoring nodes the devices uses machine learning based periodic approach for the estimation of slots requirement. For the estimated requirement, the slots are allotted for active nodes and inactive nodes remain in sleep state during their reserved slot to save energy. The sub-session2 is used for event monitoring nodes and it follows bit-map-assisted MAC protocol.

In case, if any node turns on the priority mode requirement, the system prioritizes urgent or emergency data. This mode is activated whenever critical conditions arise, such as data buffer overflow or the need for emergency data transmission. During priority mode, guaranteed reserved slots are dedicated to handling priority data, and for all other devices contention based bit-mapping approach is used for the data transmission as shown in Fig.1. In addition, the priorities are categories as given in Table 2.

**Table 2:** Priority Encoding Mode of Operation.

$BO_M$	$P_D$	$BO_C$	$E_D$	Priority	N/P
1	0	0	0	00 (Lowest)	P
X	1	0	0	01	P
X	X	1	0	10	P
X	X	X	1	11	P
0	0	0	0	XX	N

*Note: $BO_C$-Critical Buffer Overflow, $BO_M$-Moderate Buffer Overflow, $P_D$-Data Priority, $E_D$-Emergency Data, N/P-Normal/Priority Mode, X-Don’t Care

The priority encoding is used with don’t care condition (X) to define the priority. The system operates between Normal Mode and Priority Mode based on sensor requirement in particular session. When there is a moderate buffer overflow ($BO_M = 1$, buffer usage between 50% and 75%) and no other critical factors like data priority, critical buffer overflow, or emergency data, the system operates in Priority Mode but with the lowest urgency (priority level 00). As conditions change, such as the presence of prioritized data ($P_D = 1$), the system moves to a higher priority (01), ensuring that important data gets handled promptly.

When the buffer exceeds 75% usage ($BO_C = 1$, critical overflow), or when emergency data ($E_D = 1$) arrives, the system further elevates its urgency. A critical buffer overflow leads to high priority (10), and the presence of emergency data immediately sets the system to the highest priority level (11). These conditions force the system into Priority Mode, ensuring that critical or emergency situations are addressed to prevent data loss or system failure.

As already discussed the proposed method provides two-fold benefits. In the normal mode, it saves energy more efficiently, and in priority mode, it provides guaranteed delivery of data in an energy-efficient manner with low latency. To analyze the performance of the proposed, we have derived the mathematical model for two different conditions. The first scenario considers that all the sensor devices are homogeneous, and the protocol works only in normal mode. This protocol is named the Periodic approach for Hybrid Medium Access Control (PaHMAC) protocol. In the second scenario, it is considered that the devices are heterogeneous, and some devices generate critical data that needs priority transmission. This protocol is called the Priority-aware Periodic approach for the Hybrid Medium Access Control ($Pa^2HMAC$) protocol. The mathematical model of both *PaHMAC* and $(Pa)^2HMAC$ are discussed in the next section.

## Mathematical model of *PaHMAC* and $Pa^2HMAC$

In this section, the mathematical model of the proposed protocol is derived for PaHMAC and $Pa^2HMAC$. Algorithm 1 is proposed for the optimal periodicity estimation of the proposed method. The symbols used for the derivation are described in Table 3. For the proposed scenario, the network devices are categorized into continuous, periodic, and event monitoring devices. Overall there are total *m* continuous monitoring and periodic monitoring nodes and $m_1$ nodes are continuous monitoring nodes. Thus, there are total $m-m_1$ periodic monitoring nodes. However, in a particular session only few periodic monitoring will be active based on the periodicity and type of sensor. The $m_2$ nodes are considered as active periodic nodes in a particular session which depends on periodic data generation probability ($p_p$). The equation of active periodic monitoring nodes ($m_2$) is given below:

1 $$\begin{aligned} m_2 = (m-m_1)\times p_p \end{aligned}$$

The total sensor nodes count is *N* and thus, the total event monitoring nodes are $N-m$. The total active event monitoring nodes in particular session depends upon event probability ($p_e$). The equation of active event monitoring nodes ($m_2$) is given below:

2 $$\begin{aligned} n = (N-m)\times p_e \end{aligned}$$

For the priority mode, the total probability of priority is represented as $\Phi$. As already discussed that the priority is categorized into 3 categories, buffer overflow probability ($\Phi _{BO}$), data priority probability ($\Phi _{DP}$), and emergency data probability ($\Phi _{ED}$) as given below.

3 $$\begin{aligned} \Phi = \Phi _{BO}+\Phi _{DP}+\Phi _{ED} \end{aligned}$$

In the priority mode, the priority nodes transmit data using guaranteed reserved slots and all other nodes transmit data based on bit-mapping based reservation as shown in Fig. 1. Thus, the guaranteed reserved slots and remaining active event-monitoring slots are estimated based on below given Eq. 4, and Eq. 5 .

4 $$\begin{aligned} m_0=N\times \Phi = N\times (\Phi _{BO}+\Phi _{DP}+\Phi _{ED}) \end{aligned}$$

5 $$\begin{aligned} n_0=(N-m_0)\times p_e=N(1-\Phi )\times p_e \end{aligned}$$

**Algorithm 1:**
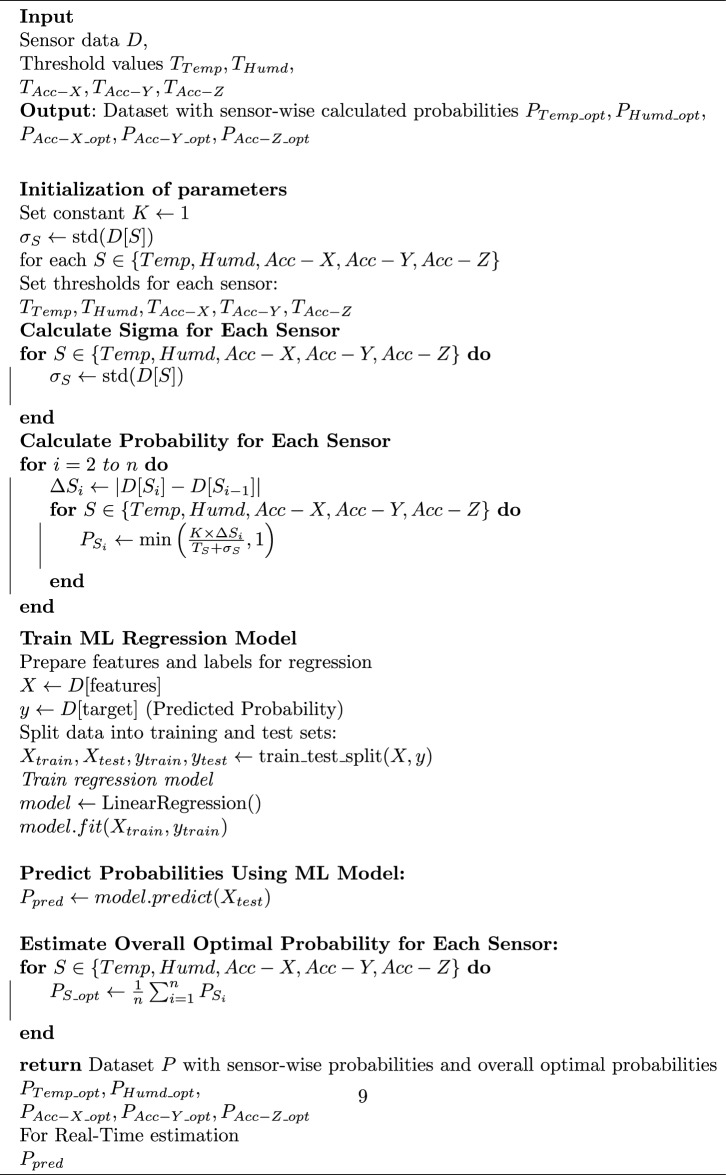
Sigma Estimation and Sensor-Wise Optimal Probability Calculation

In the proposed work, machine learning based approach is used for the estimation of early prediction and allotment of optimal slots. The optimal transmission probability is estimated based on the dataset training and testing. For the training, three different sensors (Temperature, Humidity and Acceleration Sensor) data are used as given in^30^.

**Table 3:** Parameter and Notation.

Notation	Quantity
$m_1$	No. of Continuous Monitoring (CM) Nodes
$m_2$	No. of Active Periodic Monitoring (PM) Nodes
*m*	Total number of CM and PM Nodes
*N*	Total Number of Sensor Nodes
$\Phi$	The Sum of Emergency Data, Priority Data, and Buffer Overflow Probabilities
$\Phi _{BO}$	Buffer Overflow Probability
$\Phi _{DP}$	Data Priority Probability
$\Phi _{ED}$	Emergency Data Probability
$m_0$	Number of Buffer Overflow, Emergency Data, and Data Priority Nodes
$n_0$	Number of Active Event Monitoring Nodes
$p_p$	Periodic Data Generation Probability
$p_e$	Event Generation Probability
$P_{Temp\_opt}$	Optimal probability of Temperature Sensor
$P_{Humd\_opt}$	Optimal probability of Humidity Sensor
$P_{Acc\_X\_opt}$	Optimal probability of Acceleration Sensor (X)
$P_{Acc\_Y\_opt}$	Optimal probability of Acceleration Sensor (Y)
$P_{Acc\_Z\_opt}$	Optimal probability of Acceleration Sensor (Z)
$\kappa$	Probability Scaling
$\Delta S_i$	Deviation in between consecutive readings
$T_S$	Threshold of Sensor Reading
$\sigma$	Standard Deviation of overall sensor readings

For the estimation of optimal probability, Algorithm 1 is proposed. The algorithm starts with the initialization of the parameters. The $\sigma _S$ is estimated based on the standard deviation of sensor and thresholds for sensor, including temperature, humidity, and accelerometer readings in the X, Y, and Z directions. The standard deviation ($\sigma _S$) for each sensor’s data is calculated to estimate the variability in sensor readings. The value of $\sigma _S$ is essential to compute sensor-wise probabilities, which indicate the likelihood of a significant change in sensor readings, based on predefined thresholds for each sensor type.

Next, the algorithm calculates the sensor-wise probabilities. For each training data sample *i*, the absolute change in sensor data ($\Delta S_i$) is computed, and a probability $P_{S_i}$ is determined by comparing the change with the threshold and standard deviation as given in Eqs. 6 and 7.

6 $$\begin{aligned} P_{Temp\_opt}=\left[ \kappa \times \frac{\Delta S_i}{T_S+\sigma }\right] _{i=1}^{i=n} \end{aligned}$$

7 $$\begin{aligned} \Delta S_i = S_{i}-S_{i-1} \end{aligned}$$

This process ensures that the probabilities reflect both the magnitude of change and the sensor’s time-series training data variation. At the end, a machine learning regression model is trained to predict the probabilities based on the sensor data features (e.g., mean and standard deviation). The model uses training and test datasets to fit a linear regression model and then predicts the probabilities for the test data.

For the mathematical estimation of overall energy consumption of the proposed model, the algorithm computes an overall optimal probability ($P_{S\_opt}$) for each sensor by averaging the predicted probabilities over time. The optimal probability is used for the estimation of energy consumption of the network. For the performance analysis we have developed a two mathematical model. In the first model, we have considered only normal mode transmission and in the second mode, both priority and normal mode transmissions are considered. The mathematical model of both the scenarios are derived below.

### Mathematical model of periodic approach for hybrid medium access control (PaHMAC)

The setup phase energy consumption is almost negligible w.r.t. to steady state, therefore, setup phase energy consumption is ignored for all the protocols. The Eq. 8 equation represents the energy consumption during the Post-Setup Phase (PSP). Each sensor transmit a control packet in sub-slot to represent the type of sensor (continuous/periodic/event). During each sub-slot, each sensor node transmit data and remaining $N-1$ nodes remain in idle mode. At the end of post-set phase the cluster-head broadcasts a control packet $T_{CH}$. *N* sensor nodes receives data packet and therefore total energy consumption is $NP_{Rx}T_{CH}$. The overall, energy consumption of post setup phase is given below in Eq. 8:

8 $$\begin{aligned} & EC_{PSP} = P_{Tx} T_{CH} + NP_{Rx} T_{CH}\nonumber \\ & +NP_{Rx}T_C+N\bigg (P_{Tx}T_C+(N-1)P_IT_C\bigg ) \end{aligned}$$

The energy consumption during subsession 1 ($EC_{SS1}$) is described in Eq. 9. During subsession-1, all continuous monitoring nodes ($m_1$) and active periodic monitoring nodes ($m_2$) transmit the data in their reserved slot and all other SNs remain in sleep mode.

9 $$\begin{aligned} EC_{SS1} = m_1P_{Tx}T_D+m_2P_{Tx}T_D+m_1P_{Rx}T_D+m_2P_{Rx}T_D \end{aligned}$$

The subsession 2 is categorized into Contention Access Period (CAP) and contention free period (CFP). The Eq. 10 corresponds to the energy consumption in subsession 2 during the CAP. During CAP, all the event monitoring active nodes (*n*) transmits the control message in sub-slots. During each subslot one SN transmits the control message and remaining $N-m-1$ nodes remain in idle mode ($P_I$). All remaining inactive event monitoring nodes $N-m-n$ remain in inactive mode for full CAP duration.

10 $$\begin{aligned} \begin{aligned} EC_{SS2-CAP} =&P_{Tx}T_{CH}+(N-m)P_{Rx}T_{CH}+nP_{Rx}T_C\\ &+n\bigg (P_{Tx}T_C+(N-m-1)P_IT_C\bigg )\\&+(N-m-n)\bigg (P_IT_C+(N-m-1)P_IT_C\bigg ) \end{aligned} \end{aligned}$$

The second part of subsession 2 is Contention Free Period (CFP). During CFP, the active event monitoring nodes transmit data to the CH node as given in Eq. 11.

11 $$\begin{aligned} \begin{aligned} EC_{SS2-CFP} =&nP_{Tx}T_{D}+nP_{Rx}T_{D} \end{aligned} \end{aligned}$$

The total energy consumption for the *PAHMAC* protocol ($EC_{PAHMAC}$), can be obtained by combining all the phases. It sums the energy consumption from the Post-Setup Phase ($EC_{PSP}$), subsession 1 ($EC_{SS1}$), subsession 2 during the Contention Access Period ($EC_{SS2-CAP}$), and subsession 2 during the Contention Free Period ($EC_{SS2-CFP}$). The final total energy is scaled by a number of sessions *K* as given in Eq. 12.

12 $$\begin{aligned} \begin{aligned} EC_{PAHMAC} =&EC_{PSP}+K\bigg (EC_{SS1}+EC_{SS2-CAP}+EC_{SS2-CFP}\bigg ) \end{aligned} \end{aligned}$$

### Mathematical model of priority-aware periodic approach for the hybrid medium access control ($Pa^2HMAC$)

In this scenario, we have considered both normal mode as well as priority mode of operations. There are total *K* sessions and the sessions normal/priority mode sessions ($K_N$ and $K_{PM}$) are estimated based on the priority mode probability ($\Phi$) as given in Eq. 13.

13 $$\begin{aligned} \begin{aligned} K=&K_N+K_{PM}=K\times \Phi +K\times (1- \Phi ) \end{aligned} \end{aligned}$$

The post setup phase of priority aware mode is equivalent to the scenario 1 (Normal Mode). Thus , Eq. 14 is equivalent to Eq. 8.

14 $$\begin{aligned} \begin{aligned} EC_{PAPSP} =&P_{Tx} T_{CH} + NP_{Rx} T_{CH}+NP_{Rx}T_C\\&+N\bigg (P_{Tx}T_C+(N-1)P_IT_C\bigg ) \end{aligned} \end{aligned}$$

For this hybrid mode of operation, the CH broadcasts a control message to inform all the nodes about mode of operation. The energy consumption is given below in Eq. 15.

15 $$\begin{aligned} \begin{aligned} EC_{PANP}^{NM} =&P_{Tx} T_{CH} + NP_{Rx} T_{CH} \end{aligned} \end{aligned}$$

Now we will consider one session for normal mode and one for priority mode operation as shown in Fig. 1. Eqs. 16, 17,18 and 19 are normal mode of operation and the energy consumption in this mode is equivalent to the normal mode operation. Thus, the Eq. 16 ,17 and 18 is equivalent to Eqs. 9, 10 and 11.

16 $$\begin{aligned} & EC_{PASS1}^{NM} = m_1P_{Tx}T_D+m_2P_{Tx}T_D+m_1P_{Rx}T_D+m_2P_{Rx}T_D \end{aligned}$$

17 $$\begin{aligned} & EC_{PASS2-CAP}^{NM} = P_{Tx}T_{CH}+(N-m)P_{Rx}T_{CH}\nonumber \\ & +nP_{Rx}T_C+n\bigg (P_{Tx}T_C+(N-m-1)P_IT_C\bigg )\nonumber \\ & +(N-m-n)\bigg (P_IT_C+(N-m-1)P_IT_C\bigg ) \end{aligned}$$

18 $$\begin{aligned} & EC_{PASS2-CFP}^{NM} = nP_{Tx}T_{D}+nP_{Rx}T_{D} \end{aligned}$$

The overall energy consumption for the normal mode cycles ($K_N$) can be estimated based on Eqs. 15 ,16, 17 and 18 as given in Eq. 19.

19 $$\begin{aligned} \begin{aligned} EC_{{PA}^2HMAC}^{NM} =&K_N\bigg (EC_{PANP}^{NM}+EC_{PASS1}^{NM}+EC_{PASS2-CAP}^{NM}\\&+EC_{PASS2-CFP}^{NM}\bigg ) \end{aligned} \end{aligned}$$

The second mode of operation is priority mode of operation which is shown for session-j. Similar to the normal mode, in this mode also, the session starts with broadcast message as derived in Eq. 15.

20 $$\begin{aligned} \begin{aligned} EC_{PANP}^{PM} =&P_{Tx} T_{CH} + NP_{Rx} T_{CH} \end{aligned} \end{aligned}$$

In the priority mode of operation, the session is divides into 2 sub-sessions. The first sub-session consist of guaranteed time slots ($m_0$) as derived in Eq. 4. The total energy consumption in sub-session1 can be estimated as given in Eq. 21.

21 $$\begin{aligned}&EC_{PASS1}^{PM} =&m_0P_{Tx}T_D+m_0P_{Rx}T_D \end{aligned}$$

22 $$\begin{aligned}&EC_{PASS2-CAP}^{PM} = P_{Tx}T_{CH}+(N-m_0)P_{Rx}T_{CH}\nonumber \\&+n_0P_{Rx}T_C+n_0\bigg (P_{Tx}T_C+(N-m_0-1)P_IT_C\bigg )\nonumber \\&+(N-m_0-n_0)\bigg (P_IT_C+(N-m_0-1)P_IT_C\bigg ) \end{aligned}$$

The second sub-session follows bit-mapping approach for the data transmission. All the remaining nodes are considered as event monitoring nodes and the active event monitoring nodes is derived in Eq. 5. Thus, the total energy consumption during CAP of sub-session2 can be estimated as explained in Eq. 17. The total energy consumption in CAP is given in Eq. 22.

The second part of sub-session2 is CFP and during this period each active event monitoring node transmit the data to their allotted time slot as given in Eq. 23.

The total sum of energy consumption of overall priority mode sessions ($K_{PM}$) is sum of Eqs. 20 ,21 , 22 and 23 as given in Eq. 24.

23 $$\begin{aligned} EC_{PASS2-CFP}^{PM} =&n_0P_{Tx}T_D+n_0P_{Rx}T_D \end{aligned}$$

24 $$\begin{aligned} EC_{{PA}^2HMAC}^{PM} =&K_{PM}\bigg (EC_{PANP}^{PM}+EC_{PASS1}^{PM}+EC_{PASS2-CAP}^{PM}\nonumber \\&+EC_{PASS2-CFP}^{PM}\bigg ) \end{aligned}$$

Thus, the overall sum of energy consumption for *K* sessions can be derived from Eq. 14, Eq. 19, and Eq. 24. The total energy consumption for $K=K_N+K_{PM}$ sessions is given by Eq. 25.

25 $$\begin{aligned} \begin{aligned} EC_{{PA}^2HMAC} = EC_{PAPSP}+EC_{{PA}^2HMAC}^{NM}+EC_{{PA}^2HMAC}^{PM} \end{aligned} \end{aligned}$$

The performance of the proposed method is compared for two different scenarios (*PaHMAC* and $Pa^2HMAC$). The *PaHMAC* consist of only normal mode of operation. The energy consumption of *PaHMAC* is derived in Eq. 12. The $Pa^2HMAC$ consist of both normal mode and priority mode of operation. The energy consumption of $Pa^2HMAC$ is derived in Eq. 25. The result analysis of both methods is discussed in next section.

## Result analysis and discussion

The periodic transmission probability is estimated based on the Algorithm 1 plays a critical role in overall energy consumption of a wireless sensor network. In addition, the priority service for various conditions also affects overall energy consumption of the network. Therefore, in the present work, the overall energy consumption is estimated for only normal operation like other existing methods, i.e., *PaHMAC*, and also analyzed for priority aware mode, i.e., $Pa^2HMAC$. Priority aware mode provides critical data transmission service with minor comprise in energy consumption. In the present work, we have derived the expression of the proposed methods, *PaHMAC* and $Pa^2HMAC$, and the performance is compared with the existing methods, namely ASHMAC, E-BMA, EA-TDMA and TDMA using the same simulation environment background and parameters^4^.

The data traffic for sensor nodes is used based on a dataset provided in^30^. The simulation is executed for 100 sessions. For the performance analysis, 2500 samples are utilized for each sensor to estimate the periodic data transmission of the nodes. MATLAB simulation is employed as the simulator for the analysis. It is assumed that the network utilizes a 2.4 GHz mote module enabled with a CC2420 radio (standard protocol of IEEE 802.15.4) to analyze energy usage. The power consumption rating for CC2420 RF is 50 mW for transmission, 54 mW for reception, and 50 mW for standby listening mode (idle mode). The data rate of IEEE 802.15.4 MAC for WPAN operation using ZigBee is 250 kbps. However, in the present work a worst-case scenario is considered as reported in other works. Therefore, the practical data rate is set at 25 kb/s (assumed value). The control packet size is fixed at 5 Bytes and default data packet size is 100 Bytes. The performance is analyzed in terms of energy consumption and latency. For the performance comparison, the number of sensor nodes, data packet size, number of rounds, number of continuous monitoring nodes, and event generation probability is varied for different cases. The default parameter for the simulation analysis are discussed in Table 4.

**Table 4:** Simulation Parameter Values.

Parameter	Quantity	Value
$\kappa$	Scaling factor	1
$\sigma _T$	Estimated value of Temperature Sensor Variance	0.820
$\sigma _H$	Estimated value of Humidity Sensor Variance	0.045
$\sigma _{Ax}$, $\sigma _{Ay}$, $\sigma _{Az}$	Estimated value of Accelerator Sensor Variance	0.00417, 0.11425, 0.00499
$T_{T}$, $T_{H}$, $T_A$	Thresholds of Temperature, Humidity, and Accelerator Sensor	5, 2, 0.01
*K*	Number of Rounds	100
$\Phi _{BO}$	Buffer Overflow Probability	0.2
$\Phi _{DP}$	Data Priority Probability	0.1
$\Phi _{ED}$	Emergency Data Probability	0.1
$p_p$	Estimated value of Periodic Data Generation Probability	0.2
$p_e$	Event Generation Probability	0.2
$P_{Tx}$	Transmitter Power Consumption	50 mW^4^
$P_{Rx}$	Receiver Power Consumption	54 mW
$P_{I}$	Idle State Power Consumption	50 mW
$T_{D}$	Data packet transmission time	100 Bytes/Practical Data Rate
$T_{C}$	Control packet transmission time	5 Bytes/Practical Data Rate
$T_{CH}$	CH broadcast control packet transmission time	10 Bytes/Practical Data Rate

### Case 1: number of nodes

**Fig. 2:**
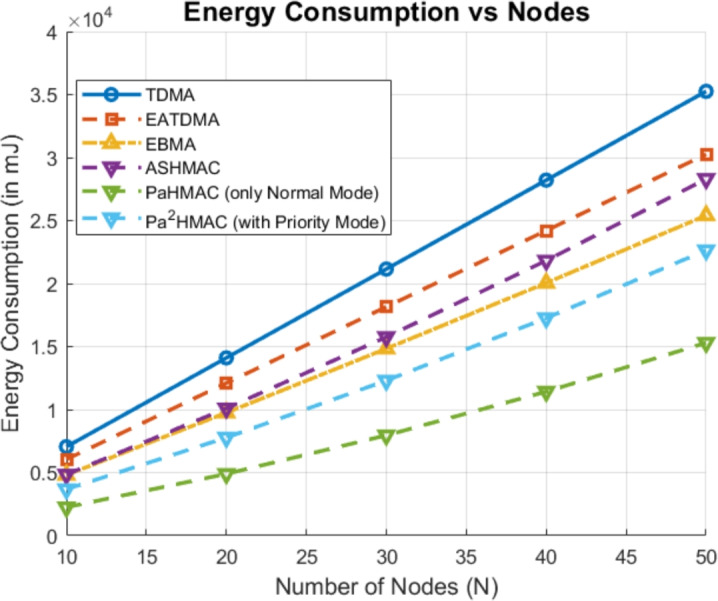
Energy Consumption Comparison of MAC Protocols as Node Count Increases.

In the first case, the number of nodes are varied from 10 to 50. The results are analyzed for the proposed method for normal mode operation as well as hybrid mode operation (includes both normal and priority mode). As shown in Fig. 2, TDMA exhibits the highest energy consumption, with its energy usage increasing significantly as the number of nodes increases.

EATDMA and ASHMAC follow similar behavior, also shows a steady rise in energy consumption, but with slightly lower values than TDMA. EBMA demonstrates improved energy efficiency and consumes less energy in comparison to the other protocols. However, the proposed *PaHMAC* (Normal Mode) and *Pa*²*HMAC* (Hybrid Mode) shows the lowest energy consumption, indicating that these protocols are the most energy-efficient, particularly as the number of nodes increases. The normal mode energy consumption of proposed method is much better in terms of energy consumption with respect to *Pa*²*HMAC* (hybrid mode). The results showcases that the proposed method performs much better which makes it ideal for scenarios requiring both low energy usage and high scalability.

**Fig. 3:**
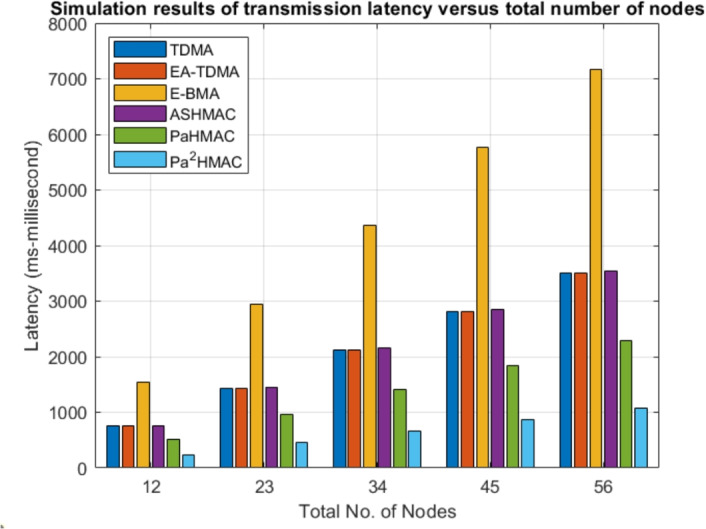
Maximum Transmission Latency Comparison of MAC Protocols as Node Count Increases.

The maximum transmission latency is also an important and critical factor need analysis to showcase the superiority of the proposed method. The main objective of the proposed protocol is to reduce energy consumption with out compromising with maximum transmission latency. Therefore, the maximum transmission latency is also analyzed for the varying number of nodes. As shown in Fig 3, TDMA has relatively high maximum transmission latency, particularly as the number of nodes grows, reaching almost 7000 ms for 56 nodes, indicating challenges in scalability. EA-TDMA performs slightly better but still experiences significant latency, especially in larger networks. E-BMA shows the highest latency across most node counts, suggesting inefficiency in handling increased traffic. The only advantage of E-BMA is that it consumes with respect to the other variants of MAC. The ASHMAC protocol saves energy without compromising with latency. In contrast, ASHMAC, *PaHMAC*, and *Pa*²*HMAC* demonstrate much lower maximum transmission latency, with *Pa*²*HMAC* consistently having the lowest across all node counts, making it the most efficient for reducing transmission delays in larger networks. The beauty of the proposed method is that in purely normal mode has very low energy consumption but slightly higher latency. But the hybrid mode is more suitable for real-time critical data transmission but slightly less energy-efficient. This highlights *Pa*²*HMAC* as a strong candidate for applications requiring minimal latency.

### Case 2: event generation probability

**Fig. 4:**
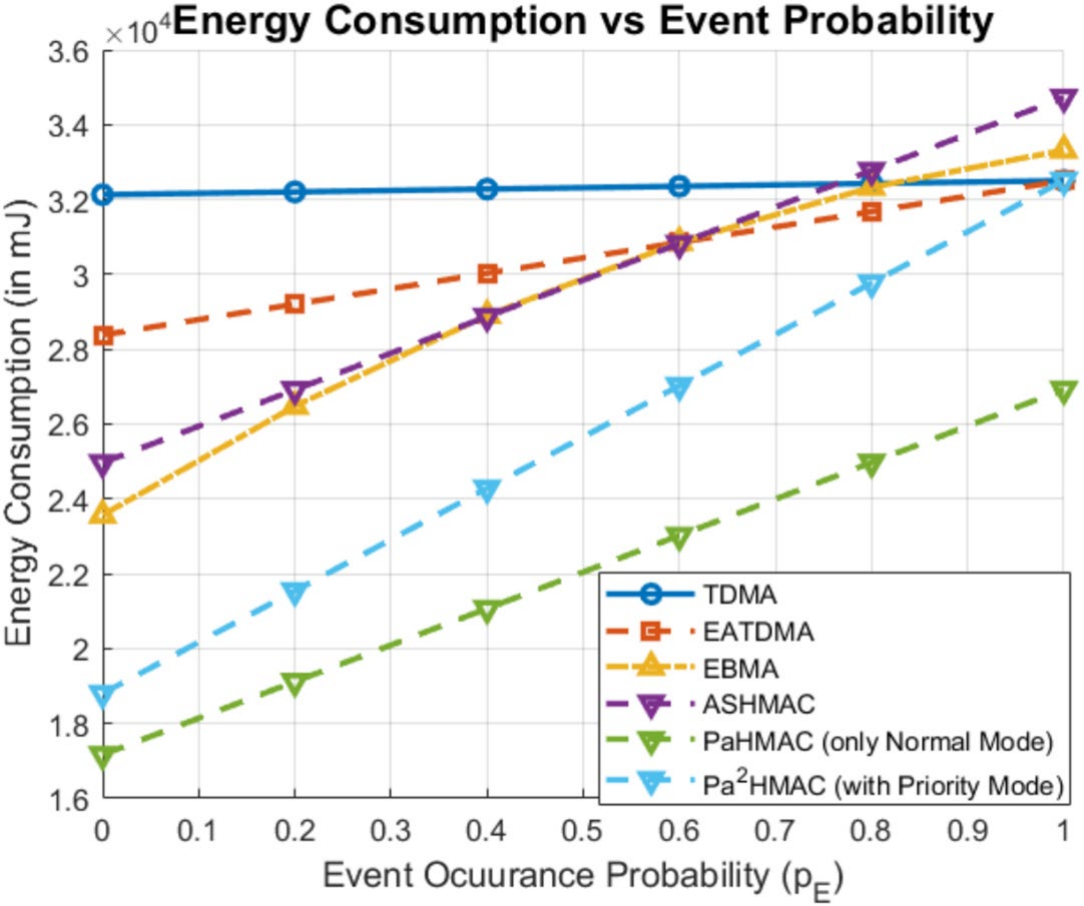
Energy Consumption Analysis of MAC Protocols Based on Event Occurrence Probability.

As already discussed, the event generation probability can vary from 0 to 1 based on current data traffic. As shown in Fig. 4, the event probability increases, TDMA maintains a nearly constant high energy consumption which makes it inefficient in energy use across all probabilities. EATDMA and EBMA show increasing energy consumption with higher event probabilities, but at a slightly lower rate than TDMA. ASHMAC shows better energy efficiency, although its energy consumption also rises with event probability. *PaHMAC* and $Pa^2HMAC$ (with priority mode) consumes lower energy consumption. $Pa^2HMAC$ demonstrates the most efficient performance, especially as event probability increases. This highlights $Pa^2HMAC$ as the most energy-efficient protocol in dynamic event-driven scenarios.

### Case 3: continuous monitoring nodes

**Fig. 5:**
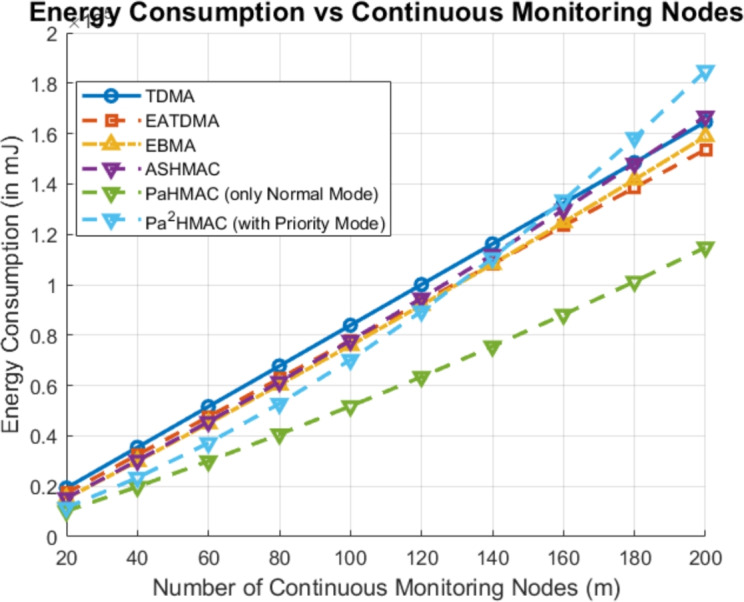
Energy Consumption Analysis Based on the Number of Continuous Monitoring Nodes.

To further analyze the dynamic traffic of continuous monitoring nodes, the number of continuous monitoring nodes increases from 20 to 200 as shown in Fig 5. In this case also, TDMA shows the highest energy consumption, which increases with the node count. The EATDMA and EBMA also exhibit high energy usage but are more efficient than TDMA. In contrast, *PaHMAC* (Normal Mode) demonstrates the lowest energy consumption, indicating its superior energy efficiency, while $Pa^2HMAC$ (Priority Mode) and ASHMAC show moderate performance in this case. From the result, we can conclude that the $Pa^2HMAC$ offers a good balance between energy savings and priority handling. *PaHMAC* is most energy-efficient protocol for continuous monitoring, particularly as the number of nodes increases.

### Case 4: packet size

**Fig. 6:**
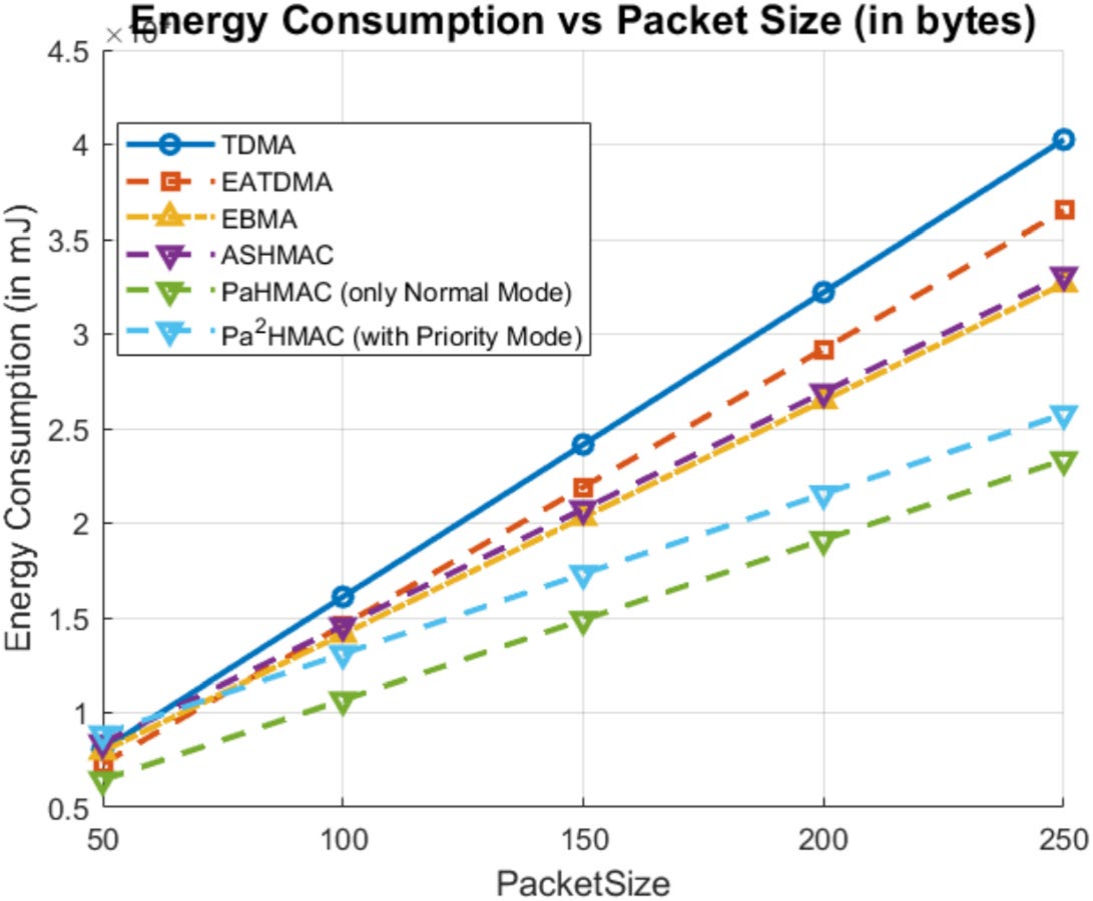
Energy Consumption Analysis Based on Packet Size.

**Fig. 7:**
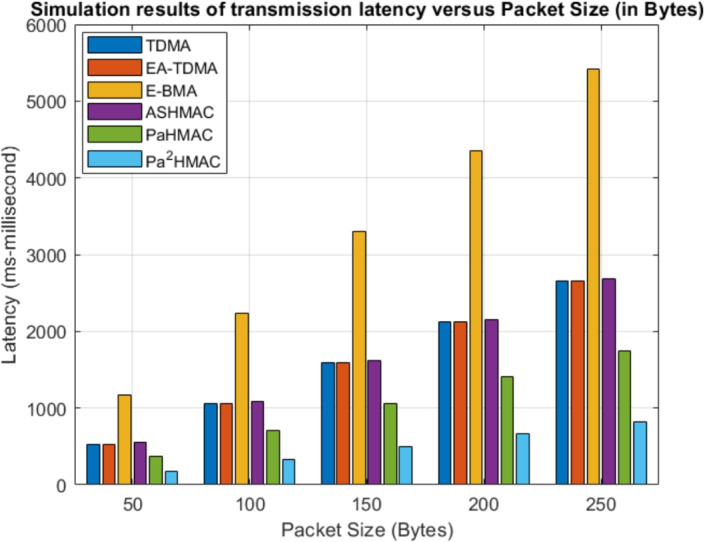
Maximum Transmission Latency Analysis Based on Packet Size.

To further analyze the performance, the packet size is varied from 50 Bytes to 250 Bytes as shown in Fig. 6. Like other parameters, the energy consumption of the MAC protocol increases with packet size. The energy consumption of the proposed method is much lesser in normal mode. However, in hybrid mode the energy consumption is similar to the E-BMA and ASHMAC. In hybrid mode, the proposed method compromises with energy consumption but the maximum transmission latency of the hybrid mode ($Pa^2HMAC$) is minimal and it provides guaranteed delivery of critical data packets in real time as shown in Fig. 7.

### Case 5: number of rounds

**Fig. 8:**
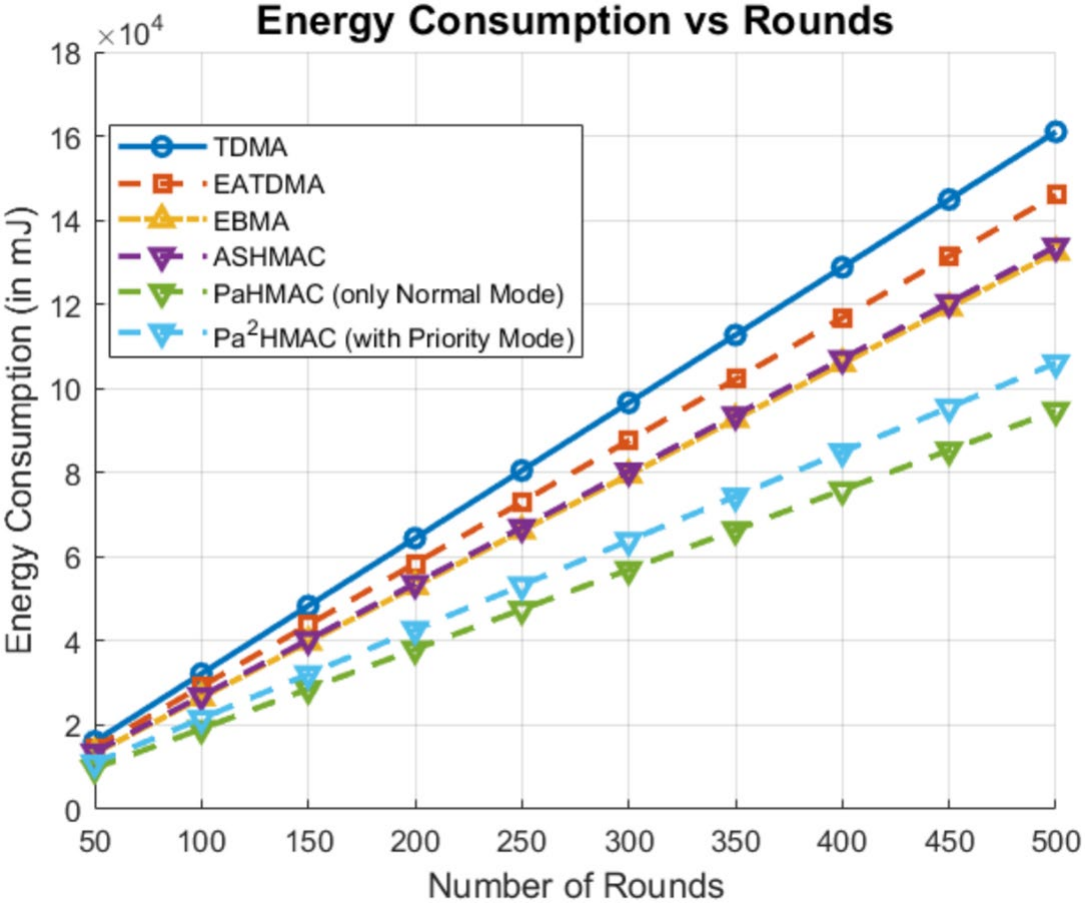
Energy Consumption Analysis Based on Number of Rounds.

As shown in Fig. 8, the performance of TDMA, EATDMA, E-BMA, ASHMAC, *PaHMAC* in normal mode, and $Pa^2HMAC$ in priority mode is compared in terms of energy consumption over increasing rounds. The results show same patter as reported in previous cases. The TDMA consumes high energy, while EATDMA and EBMA show moderate energy consumption. ASHMAC is more energy efficient than these, but *PaHMAC* and $Pa^2HMAC$ show the lowest energy usage, with *PaHMAC* (Normal Mode) being the most efficient overall. This also indicates that *PaHMAC* protocols are highly energy-efficient, making them suitable for energy-sensitive applications.

## Conclusion

In the present work, an energy-efficient periodicity and priority aware MAC protocol is proposed, i.e., *PaHMAC* and $Pa^2HMAC$. The proposed protocols demonstrate the significant improvements in terms of energy efficiency compared to existing MAC protocols such as TDMA, EATDMA, EBMA, and ASHMAC. The ASHMAC transmits data with low latency. However, consumes higher energy with respectr to E-BMA. Therefore, in the present periodicity aware MAC method is proposed which determines optimal periodicity. The simulation analysis show that *PaHMAC* (Normal Mode) offers the lowest energy consumption with nominal latency, making it an ideal choice for scenarios requiring efficient energy usage. The $Pa^2HMAC$ (Priority Mode) provides priority service to the critical nodes without much compromising with latency. The results prove that $Pa^2HMAC$ also performs exceptionally well, providing a balanced trade-off between energy efficiency and priority-based transmission for critical data, ensuring minimal latency. In the proposed method, this is achieved by estimation of optimal periodicity which makes it highly suitable for real-time applications where timely data delivery is crucial.

To prove the superiority, the performance of the proposed method is compared across various cases, including the number of nodes, event generation probability, continuous monitoring nodes, and packet size. The results clearly highlights the superiority of the proposed methods. The results proves that the proposed methods *PaHMAC* and $Pa^2HMAC$ protocols not only reduces the energy consumption but also effectively handle latency, particularly in larger networks or critical event-driven scenarios. The hybrid mode of $Pa^2HMAC$ proves especially advantageous for real-time data transmission with guaranteed delivery. The added features make the proposed protocols highly adaptable for diverse wireless sensor network environments, offering both scalability and energy efficiency.

In future work, we plan to implement dynamic estimation of priority metrics using lightweight adaptive learning models. Furthermore, a cross-layer approach will be explored to enhance overall system security and responsiveness by enabling better coordination between MAC and network layers.

## Data Availability

The datasets used in this study are publicly available and can be accessed through the below-mentioned links. Machitje, Motlalepule (2024). The vibration sensor on railway lines. University of Pretoria. Dataset. https://doi.org/10.25403/UPresearchdata.24973911.v1.
